# Intraoperative irreversible electroporation for margin accentuation in locally advanced intrahepatic cholangiocarcinoma: a case series

**DOI:** 10.3389/fsurg.2026.1835187

**Published:** 2026-06-29

**Authors:** Martin Brichard, Claude Bertrand, Quentin Gilliaux, Bruno Krug, Hadrien Fourneau, Claude Schalbar, Alexandra Dili

**Affiliations:** 1 Department of Surgery, CHU-UCL Namur Site Godinne, Namur, Belgium; 2 Department of Oncology, CHU-UCL Namur Site Godinne, Namur, Belgium; 3 Department of Nuclear Medicine, CHU-UCL Namur Site Godinne, Namur, Belgium; 4 Department of Interventional Radiology, CHU-UCL Namur Site Godinne, Namur, Belgium; 5 Department of Oncology, Centre Hospitalier du Nord, Ettelbruck, Luxembourg

**Keywords:** hepatic vein, intrahepatic cholangiocarcinoma, irreversible electroporation, liver resection, locally advanced, margin accentuation, vascular involvement

## Abstract

**Introduction:**

The incidence of intrahepatic cholangiocarcinoma (iCCA) is increasing worldwide and remains associated with a poor prognosis. Surgical resection is the only potentially curative treatment; however, only a minority of patients are eligible due to advanced disease and vascular involvement. Irreversible electroporation (IRE) is a non-thermal ablative modality that enables tissue treatment while preserving vascular structures. This study aimed to evaluate the feasibility, safety, and preliminary oncologic outcomes of intraoperative IRE as an adjunct for margin accentuation in locally advanced iCCA involving critical vascular structures.

**Methods:**

Between 2023 and 2024, three patients with initially unresectable, centrally located mass-forming iCCA were treated at a single center. All tumors involved the hepatic vein of the future liver remnant, precluding conventional R0 resection. Patients underwent extended hepatectomy combined with intraoperative IRE applied at the tumor–hepatic vein interface, followed by vascular detachment to preserve venous outflow.

**Results:**

All procedures were completed as planned. No intraoperative complications or IRE-related postoperative adverse events were observed. Ninety-day mortality was 0%. Postoperative imaging confirmed preserved hepatic vein patency in all patients. At a median follow-up of 18 months (range 15–27), no local recurrence at the vascular interface was detected. One patient with initially multifocal disease developed a solitary intrahepatic recurrence at 17 months, which was successfully treated with local ablation. All patients were alive at last follow-up.

**Conclusions:**

Intraoperative IRE for margin accentuation appears to be a feasible and safe adjunct to extended hepatectomy in selected patients with locally advanced iCCA involving the hepatic vein of the future liver remnant. This anatomy-preserving strategy may expand surgical indications by enabling radical resection in cases otherwise deemed unresectable. Further studies are required to validate its oncologic benefit.

## Introduction

Intrahepatic cholangiocarcinoma (iCCA) is the second most common primary liver malignancy after hepatocellular carcinoma. Its incidence is steadily increasing worldwide, with a parallel rise in mortality (1–3). Most patients lack identifiable risk factors, limiting opportunities for surveillance, and despite advances in systemic therapy, prognosis remains poor (3, 4). Without treatment, median overall survival (OS) is approximately 5 months (5), while first-line gemcitabine–cisplatin extends median OS to only 17 months (6). The aggressive biology and relative chemoresistance of iCCA underscore the need for more effective multimodal treatment strategies (7).

Surgical resection remains the only potentially curative treatment, achieving 5-year OS rates of 30%–40% in selected patients (8–11). However, only 20%–30% of patients present with upfront resectable disease due to locally advanced or metastatic tumor burden (8, 10). Even after R0 resection, recurrence rates exceed 60%, and only a minority of patients are eligible for repeat local treatment (12).

Achieving negative surgical margins (R0) is a key determinant of long-term survival, particularly in node-negative disease (13). However, tumors involving major vascular structures, especially the hepatic veins of the future liver remnant, pose a major surgical challenge. In this setting, conventional resection may be infeasible or associated with a high risk of R1 margins. Recent data suggest that R1-vascular resections are associated with inferior oncologic outcomes, highlighting the need for strategies that improve local control at critical vascular interfaces (14).

Irreversible electroporation (IRE) is a non-thermal ablative technique that delivers high-voltage electrical pulses to induce permanent cell membrane permeabilization, triggering multiple spatially distinct modes of programmed cell death. Cell death within the ablation zone is heterogeneous: necrosis predominates adjacent to the electrodes, while necroptosis, pyroptosis, and apoptosis occur at increasing distances from the electrode interface, with apoptosis representing a peripheral rather than a dominant central mechanism (15–17). Unlike thermal ablation, IRE preserves the structural integrity of adjacent vessels and bile ducts, making it particularly suitable for tumors in proximity to major vascular structures (18, 19). Initially developed for locally advanced pancreatic cancer, IRE has demonstrated safety and feasibility in primary and secondary liver tumors (20–25). In pancreatic surgery, its intraoperative use for margin accentuation has been associated with acceptable morbidity and no procedure-related mortality (26–28).

Despite growing interest in IRE for hepatobiliary malignancies, published experience specifically in iCCA remains extremely limited. A recent systematic review and meta-analysis of IRE for hepatic tumors included 300 patients across nine studies, yet only 21 had iCCA, and no iCCA-specific subgroup analysis was performed (29). The most relevant prospective feasibility data come from Belfiore et al., who evaluated IRE in 15 patients with unresectable cholangiocarcinoma (8 iCCA and 7 perihilar) reporting no major complications and a mean overall survival of 18 months for the combined cohort; however, outcomes were not reported separately by tumor location, limiting conclusions specific to iCCA (23). Most remaining series either aggregate intrahepatic and perihilar cases without subgroup analysis or focus exclusively on perihilar disease, and recent reviews have consistently highlighted the paucity of iCCA-specific data as a critical gap in the field (30). Critically, no prior study has evaluated IRE as an intraoperative margin accentuation strategy in resectable iCCA with vascular involvement.

Given these properties and the absence of dedicated clinical data, IRE represents a particularly attractive adjunct in iCCA with vascular involvement, where achieving negative margins remains technically challenging.

The aim of this study was to report our initial experience with intraoperative IRE for margin accentuation in patients with locally advanced iCCA involving the hepatic vein of the future liver remnant. To our knowledge, this represents the first clinical series evaluating this approach.

## Materials and methods

### Study design

This retrospective study was conducted using a prospectively maintained clinical database.

### Ethics

The study protocol was approved by the institutional Ethics Committee (approval no. 120/2025). All procedures were performed in accordance with the Declaration of Helsinki. Written informed consent for surgery and data collection was obtained from all patients.

### Patients

Between 2023 and 2024, three consecutive patients with locally advanced intrahepatic cholangiocarcinoma (iCCA) with multisectoral hepatic involvement precluding conventional surgical resection, underwent radical liver resection combined with intraoperative irreversible electroporation (IRE) for margin accentuation at a single tertiary hepatopancreatobiliary (HPB) center. Patient demographics and baseline characteristics are summarized in Table 1.

**Table 1:** Baseline clinical and tumor characteristics of the three patients.

Variable	Patient 1	Patient 2	Patient 3
Tumor characteristics			
Tumor size (mm)	110	112	60
Location	IV-V-VIII + II + III	I-II-III-IV-VIII	I-II-IV-VIII
Number of nodules	3	1	1
Vascular contact	RHV	RHV	RHV
Contact length (mm)	34	22	25
Circumferential involvment (°)	90−180	< 90	90–180
Growth pattern	Mass forming	Mass forming	Mass forming
CA 19-9 (kU/L)	39.4	7.4	95.0
cTNM	cT2N0M0	cT1bN0M0	cT1bN0M0
Neoadjuvant therapy	Cisplatin-Gemcitabine-Durvalumab+SIRT	-	Cisplatin-Gemcitabine-Durvalumab
Liver characteristics			
UNDERLYING LIVER DISEASE	Steatosis	Steatosis	Steatosis
FLR (segment)	VI-VII	V-VI-VII	V-VI-VII
FLR pre (%)	36	55	48
HBS FLRF pre (%/min/m²)	1.9	3.6	2.7
FLR MODULATION TECHNIQUE	SIRT	-	-
FLR post (%)	58	-	-
HBS FLRF post (%/min/m²)	2.5	-	-

RHV, right hepatic vein; SIRT, selective internal radiation therapy; FLR, future liver remnant; HBS FLRF, hepatobiliary scintigraphy FLR function.

**Figure 1:**
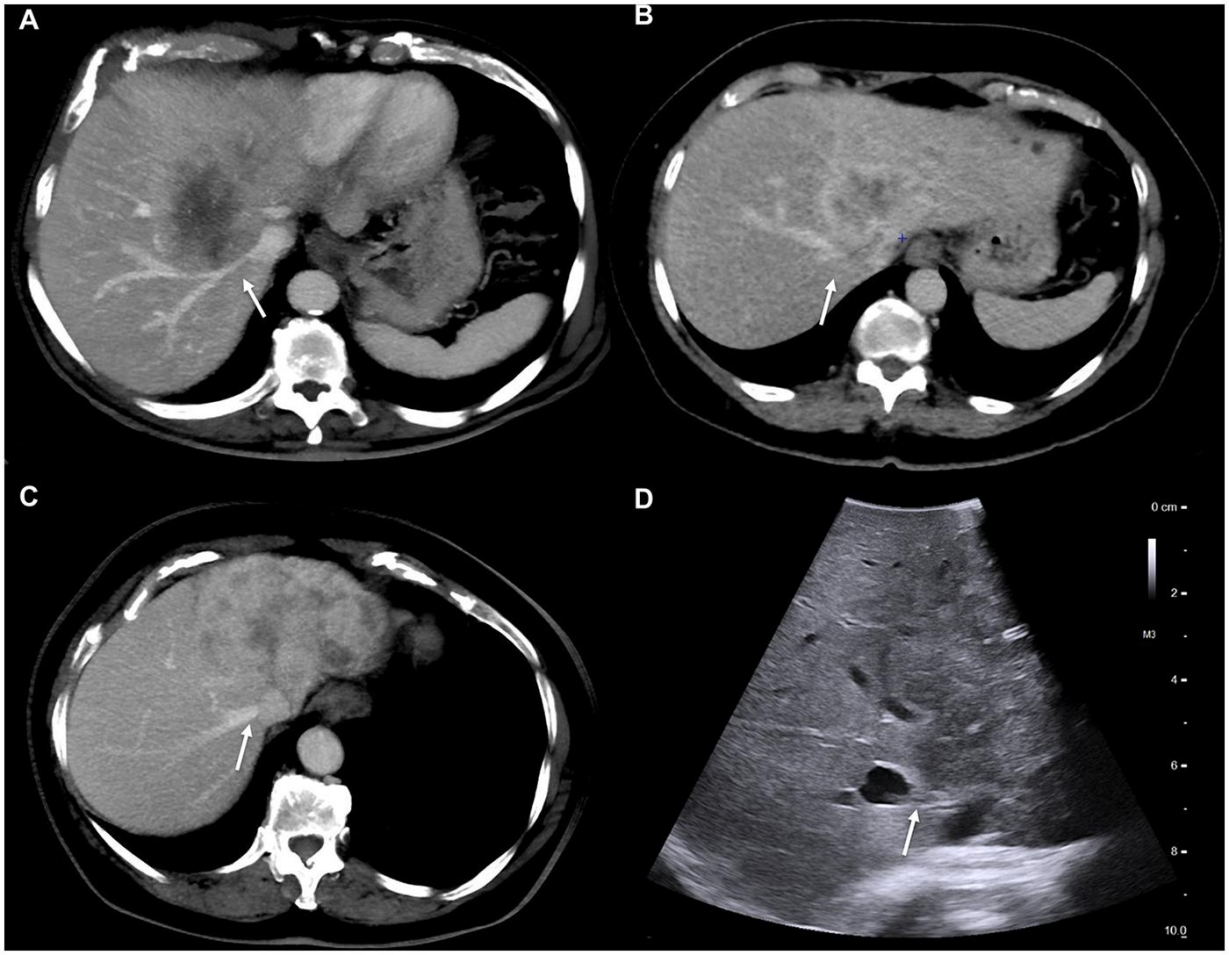
Pre-operative CT scan showing tumor contact with the right hepatic vein (white arrow) in patient 1 **(A)** and 3 **(B)**. Vascular involvement not visible in pre-operative CT scan for patient 2 **(C)** only found during intra-operative ultrasonography **(D)**.

All cases were discussed in a multidisciplinary team (MDT) setting. Tumors were considered locally advanced due to involvement of all three hepatic veins, including the hepatic vein draining the future liver remnant (FLR), precluding conventional R0 resection (Figure 1). Histological confirmation of iCCA was obtained in all patients. Tumor characteristics, including size, morphological subtype, and preoperative staging, are detailed in Table 1.

Preoperative staging included triple-phase thoracoabdominal computed tomography (CT). Additional imaging with positron emission tomography (PET)-CT and/or contrast-enhanced magnetic resonance imaging (MRI) was performed when indicated. The degree of hepatic vein involvement was assessed on preoperative cross-sectional imaging (CT and/or MRI) and characterized by two parameters: the length of tumor-hepatic vein contact (mm) and the degree of circumferential involvement, classified as less than 90°, 90–180°, or greater than 180°. These parameters are reported individually for each patient in Table 1.

FLR adequacy was assessed using functional evaluation combining volumetric analysis with 99mTc-mebrofenin hepatobiliary scintigraphy. A threshold of 2.3%/min/m² was used to define sufficient FLR function (FLRF) (31). Preoperative volumetric and functional liver data are reported in Table 1.

Two patients received neoadjuvant therapy consisting of chemo-immunotherapy, combined with selective internal radiotherapy (SIRT) in one case, for tumor downstaging and contralateral hypertrophy of the FLR. Both patients demonstrated radiological response or stable disease by RECIST criteria, however persistent hepatic vein involvement of the FLR remained. The indication for intraoperative IRE margin accentuation was determined by MDT when contralateral hepatic vein involvement was identified on preoperative imaging.

No alternative curative strategy was deemed feasible by the MDT: vascular reconstruction was considered unsuitable due to the extent of vascular involvement (>5 cm), and liver transplantation remains investigational in this setting. A vascular detachment approach combined with intraoperative irreversible electroporation for margin accentuation was therefore preferred.

### Surgical procedure

All procedures were performed by a senior HPB surgeon (AD).

Exploratory laparoscopy with intraoperative ultrasonography (IOUS) was systematically performed to exclude peritoneal or intrahepatic metastases prior to resection. Regional lymphadenectomy was carried out routinely, including frozen-section analysis of para-aortic lymph nodes (station 16) to exclude metastatic disease before proceeding with hepatectomy. Lymphadenectomy of the hepatoduodenal ligament (station 12) and common hepatic artery nodes (station 8) was subsequently completed.

IRE was performed *in situ* under IOUS guidance prior to parenchymal transection to ensure accurate needle positioning. The procedure was carried out jointly by two surgeons (AD and MB).

Parenchymal transection was then carried out under IOUS guidance, using a Cavitron Ultrasonic Surgical Aspirator (CUSA; Valleylab, Boulder, CO, USA) with intermittent pedicle clamping. Hepatic vein detachment was achieved using a combination of CUSA and blunt dissection. Vessels >2 mm were ligated with titanium clips or 3-0 sutures, and bipolar coagulation was used for hemostasis of the transection surface.

### IRE procedure

IRE was performed using the NanoKnife system (AngioDynamics, Latham, NY, USA).

**Figure 2:**
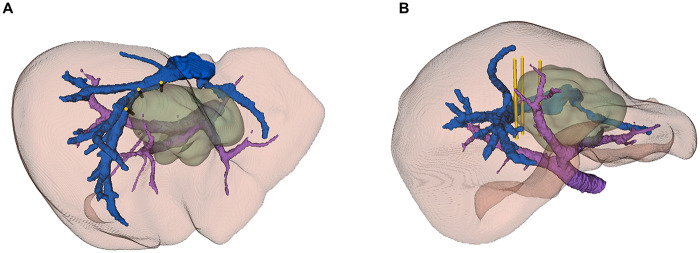
Top view **(A)** and right lateral view **(B)** of the liver showing tumor (green) contact with both hepatic veins (blue) and left portal pedicle (purple), as well as IRE needle placement (yellow). 3D rendering was generated using 3D Slicer (version 5.8.1, http://www.slicer.org/) with the RVXLiverSegmentation plugin.

Under intraoperative ultrasound (IOUS) guidance, two to three electrodes were positioned in a linear configuration along the tumor–hepatic vein interface to achieve targeted electroporation of the vascular margin (Figure 2). The active tip length was 15 mm, and inter-electrode spacing ranged from 15 to 20 mm, adjusted according to local tumor anatomy. Individual electrode number and spacing for each patient are detailed in Table 2.

**Table 2:** Perioperative outcomes of the three patients undergoing radical hepatectomy with IRE margin accentuation.

Variable	Patient 1	Patient 2	Patient 3
Surgery			
Hepatectomy	Left extended hepatectomy	Left hepatectomy extended to segment VIII with bile duct resection	Left hepatectomy extended to segment VIII
“New World” terminology	H123458 + IRE-MA	H12348-B-MHV + IRE-MA	H12348-MHV + IRE-MA
IRE needles number	3	2	2
IRE needles spacing (mm)	15	20	20
Intraoperative adverse event	Yes (RHV hemorrhage/hepatotomy)	No	No
Duration of surgery (min)	660	675	420
Pringle time (min)	10	35	20
Estimated blood loss (ml)	2,500	1,200	600
Morbi-mortality			
Morbidity (Clavien-Dindo)	IIIA	II	II
Ascitis	Yes	No	No
Bile leak	No	No	No
Post-hepatectomy hemorrhage	No	No	No
PHLF (ISGLS grade)	No	No	No
Intra-abdominal infection	Yes	No	No
Other complications	-	Delayed gastric emptying Sectorial portal vein thrombosis	Anemia Pleural effusion
ICU stay (days)	3	3	1
Hospital saty (days)	22	24	11
90-day mortality	No	No	No

IRE-MA, irreversible electroporation for margin accentuation; RHV, right hepatic vein; PHLF, post-hepatectomy liver failure; ISGLS, international study group of liver surgery; ICU, intensive care unit.

A total of 100 pulses per electrode pair were delivered at 1,500 V/cm (±300 V/cm), targeting a current of 30–40 A. The first 20 pulses were used to assess tissue conductivity, followed by 80 therapeutic pulses, in accordance with the manufacturer's protocol.

Procedural adequacy was monitored intraoperatively through continuous real-time display of delivered current by the NanoKnife generator. Amperage trends were used as a qualitative indicator of electroporation-induced tissue conductivity changes; however, given the absence of a validated current rise threshold for hepatobiliary tumors, no fixed amperage criterion was applied as a binary endpoint for procedural success (32, 33).

For larger lesions, a “pull-back” technique was used, consisting of partial electrode withdrawal followed by a second treatment cycle to ensure coverage of the superficial tumor interface.

All patients underwent cardiac synchronization using a five-lead ECG gating system to ensure pulse delivery during the cardiac refractory period and prevent arrhythmias.

### Follow up

Postoperative management followed institutional standards. Complications were recorded prospectively and graded according to the Clavien–Dindo (CD) classification and International Study Group of Liver Surgery (ISGLS) definitions (34, 35).

Patients were followed at 1 month postoperatively and every 3 months thereafter.

Adjuvant therapy consisted of capecitabine followed by chemoradiotherapy when tolerated.

### Outcomes

Clinical, perioperative, and oncological data were extracted from the institutional database.

Collected variables included patient demographics, tumor characteristics, neoadjuvant treatment, operative details, IRE parameters, histopathological findings, postoperative outcomes (morbidity, length of stay, 90-day mortality), recurrence patterns, and survival.

Disease-free survival (DFS) was defined as the interval from surgery to first radiologically confirmed recurrence. Overall survival (OS) was defined as the time from surgery to death from any cause or last follow-up.

## Results

### Peri-operative outcomes

The first patient underwent SIRT prior to surgery for tumor downstaging and to induce contralateral liver hypertrophy, given a low future liver remnant function (FLRF) of 1.9%/min/m² and the associated high risk of post-hepatectomy liver failure (PHLF). Four months after SIRT and completion of neoadjuvant therapy, FLRF increased to 2.5%/min/m². The patient subsequently underwent left extended hepatectomy [H123458 according to the “New World” terminology (36)] with IRE margin accentuation along the right hepatic vein interface. Intraoperative bleeding from the distal right hepatic vein occurred during parenchymal transection and was controlled without sequelae. No IRE-related adverse events were observed.

The second patient underwent left hepatectomy extended to segment VIII with bile duct resection. As the tumor was initially considered resectable, no neoadjuvant therapy was administered. IOUS revealed involvement of the hepatic vein of the future liver remnant (Figure 1D), prompting intraoperative IRE margin accentuation at the right hepatic vein interface. The procedure was uneventful.

The third patient underwent left hepatectomy extended to segment VIII following neoadjuvant therapy, with IRE margin accentuation at the right hepatic vein interface. No intraoperative complications occurred.

**Figure 3:**
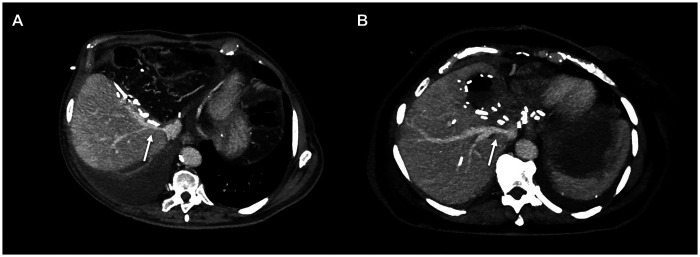
Post-operative CT scan of patient 1 **(A)** and patient 2 **(B)** at POD 7 showing good patency of right hepatic vein (white arrow).

Overall, one patient developed a major complication (Clavien–Dindo ≥ III), consisting of an intra-abdominal abscess originating from the liver section surface, treated successfully by percutaneous drainage. This complication was spatially distinct from the IRE ablation zone: IRE was performed exclusively at the hepatic vein interface and was not applied to the parenchymal section margin as a whole, which remained outside the electroporation field. No vascular complications were observed in any patient, with preserved hepatic vein patency confirmed on post-operative imaging in all cases. No patients developed PHLF, post-hepatectomy hemorrhage, bile leak, or hepatic vein thrombosis (Figure 3). 90-day mortality was 0%. Detailed perioperative characteristics are summarized in Table 2.

### Oncological outcomes

All specimens demonstrated microscopically positive margins (<1 mm) at the hepatic vein interface on final pathology.

All patients received adjuvant chemotherapy with capecitabine, followed by radiotherapy (54 Gy) to the surgical margin when clinically feasible.

One patient, initially presenting with multifocal intrahepatic disease, developed a solitary metachronous intrahepatic recurrence 17 months after surgery. This lesion was treated with microwave ablation, and the patient remains disease-free 10 months after treatment of recurrence.

No local recurrence at the hepatic vein interface was observed. All patients were alive at last follow-up (range 15–27 months). Detailed oncological outcomes are presented in Table 3.

**Table 3:** Pathological and oncological outcomes of the three patients undergoing radical hepatectomy with IRE margin accentuation.

Variable	Patient 1	Patient 2	Patient 3
Pathology			
pTNM	ypT2N0	pT2N0	ypT1bN0
Lymphadenectomy (nodes)	8	30	23
Microvascular invasion	No	Yes	No
Perineural invasion	No	Yes	No
Resection margin	R1-vascular (RHV contact)	R1-vascular (RHV contact)	R1-vascular (RHV contact)
Adjuvant therapy			
Chemotherapy	Capecitabine	Capecitabine	Capecitabine
Radiotherapy	54 Gy	54 Gy	No
Follow up			
OS (months)	27	18	15
DFS (months)	17	18	15
Local recurrence (liver margin)	No	No	No
Intrahepatic recurrence	Yes	No	No
Extrahepatic recurrence	No	No	No

RHV, right hepatic vein; OS, overall survival; DFS, disease-free survival.

## Discussion

Intrahepatic cholangiocarcinoma (iCCA) remains a highly aggressive malignancy with limited curative options. Although surgical resection is the cornerstone of treatment, long-term outcomes remain suboptimal, with 5-year OS rarely exceeding 30%–40% and recurrence rates reaching up to 70%, predominantly within the liver and frequently at or near the resection margin (37, 38). These patterns underscore the critical role of achieving adequate local disease control.

Margin status is consistently identified as one of the strongest prognostic factors in iCCA. Institutional series have reported local recurrence rates of 45%–50% after R1 resection vs. approximately 10% after R0 resection, confirming the oncologic disadvantage associated with microscopic residual disease (13). In a large multicenter cohort in which R1 was defined as ≤1 mm, overall recurrence reached approximately 65%, and R1 resection independently increased the risk of recurrence compared with R0 resection (HR 1.6) (37). Among those who recurred, 53% developed intrahepatic-only recurrence, and 24% recurred specifically at or near the surgical margin. Recurrence patterns were markedly early: 62% recurred within 12 months, and 83% within 24 months of surgery. Notably, approximately half of all margin recurrences were detected within 6 months of surgery (37). These figures underscore that the surgical margin represents a critical site of early treatment failure in iCCA, highlighting the importance of strategies aimed at improving local control in this anatomical region.

However, the surgical management of tumors involving major vascular structures, especially the hepatic vein of the future liver remnant, remains particularly challenging. In such cases, conventional R0 resection is often not feasible, and the disease is frequently deemed unresectable (14).

To overcome these limitations, increasingly aggressive surgical strategies have been developed, including vascular reconstruction, R1-vascular resections, and in highly selected cases liver transplantation. Selected series have reported encouraging outcomes, particularly in node-negative patients undergoing complex vascular procedures (11). However, these approaches are associated with increased operative complexity and morbidity, especially in cases requiring reconstruction of the last remaining hepatic vein, often necessitating extended resections, prolonged operative time, and significant blood loss (39–43).

More recently, the concept of R1-vascular resection has challenged the traditional paradigm that all R1 margins are oncologically equivalent. First described by Torzilli et al. the R1-vascular approach involves careful detachment of the tumor from major intrahepatic vessels along the Laennec capsule plane. The oncological adequacy of this approach has been previously demonstrated in both colorectal liver metastases and hepatocellular carcinoma (44–46). Milana et al. reported comparable rates of local recurrence between R0 and R1-vascular resections (16% vs. 10%). In contrast, R1-parenchymatous resection was associated with a markedly higher rate of local recurrence compared to R0 (45% vs. 10%). These findings suggest that the Laennec capsule may act as a natural barrier to tumor spread, and that vascular-adjacent margins may behave differently from true parenchymal R1 disease (39). Nevertheless, disease-free survival remains inferior in these patients (3-year DFS 11% vs. 34%), suggesting that while vascular detachment may preserve local control at the margin itself, it does not fully mitigate the overall risk of disease recurrence. This limitation highlights the potential role of adjunctive strategies such as IRE to further consolidate tumor control at the vascular interface.

Liver transplantation has recently emerged as a potential option in highly selected patients with locally advanced iCCA following successful downstaging (41, 47). However, its applicability remains limited due to strict selection criteria, donor organ scarcity, and high dropout rates. In contrast, strategies that expand resectability while preserving native liver anatomy remain broadly applicable and may benefit a larger patient population.

In parallel, multimodal strategies incorporating systemic therapy, immunotherapy, and liver-directed treatments such as selective internal radiation therapy (SIRT) have expanded the pool of patients eligible for curative-intent approaches (48–52). These modalities may enable downstaging and improve patient selection; however, they do not fully address the technical challenge of achieving adequate margins when tumors involve critical vascular structures (53). Furthermore, radiological assessment after neoadjuvant therapy remains imperfect, particularly in distinguishing fibrosis from viable tumor at vascular interfaces (54).

In this context, intraoperative irreversible electroporation (IRE) offers a complementary, anatomy-preserving approach. By inducing non-thermal cell death while preserving vascular and biliary structures, IRE enables targeted treatment of microscopic disease at sites where surgical clearance is limited. Rather than replacing resection, IRE may function as a margin-accentuation tool, specifically addressing the vascular interface where conventional techniques are either unsafe or insufficient.

This concept has been explored in other malignancies, particularly pancreatic cancer, where IRE-based margin accentuation has been associated with improved local control despite advanced disease stage and acceptable morbidity (28, 55). The IRE protocol used in this series is consistent with established NanoKnife parameters validated in preclinical porcine liver models, with expected ablation zone dimensions of approximately 2.0–2.5 cm in width and 2.5–3.0 cm in height per electrode pair position, as derived from published *in vivo* data (56, 57). Whether these findings translate to the hepatic setting remains uncertain. The anatomical context of iCCA with hepatic vein involvement differs substantially from the pancreatic resection margin. IRE at the hepatic vein interface benefits from the vessel's inherent resistance to electroporation-induced injury, which is less applicable at the pancreatic margin. Against this background, our study explores whether this approach may be extended to iCCA, a setting in which vascular proximity frequently limits resectability.

In the present series, intraoperative IRE was safely integrated into complex liver surgery, with no IRE-related complications, no vascular thrombosis, and preserved hepatic vein patency in all cases. These findings confirm the technical feasibility of applying IRE along major hepatic veins without compromising vascular integrity (18, 19).

Although all patients had microscopically positive margins at the hepatic vein interface, no local recurrence was observed during follow-up. This observation supports the hypothesis that IRE may achieve effective *in situ* tumor control that is not immediately reflected in histopathological assessment, likely due to its delayed programmed cell death mechanisms, including necroptosis, pyroptosis, and apoptosis (16, 18).

Nevertheless, these findings must be interpreted with caution. The small sample size, single-center design, and limited follow-up preclude definitive conclusions regarding oncologic benefit. Furthermore, as detailed in the Introduction, published experience with IRE specifically in iCCA remains extremely limited, with no prior study evaluating its intraoperative use for margin accentuation in the resectable setting, a knowledge gap that precludes meaningful benchmarking of our findings against historical IRE efficacy data in this disease. In addition, the incremental value of IRE over contemporary R1-vascular strategies remains uncertain, particularly in light of recent data suggesting comparable local control between R0 and R1-vascular resections (39).

Despite these limitations, this study provides the first clinical evidence that intraoperative IRE can be safely used as an adjunct to liver resection for margin accentuation in iCCA. This approach may represent a novel strategy to expand surgical indications in patients with tumors involving critical vascular structures, particularly when conventional resection or reconstruction is not feasible.

Further prospective studies are warranted to better define the oncologic impact of this strategy and to identify the patients most likely to benefit from its application.

## Conclusion

To our knowledge, this is the first case series reporting the use of intraoperative IRE for margin accentuation in locally advanced iCCA.

In this preliminary experience, intraoperative IRE was feasible and safe when applied at the hepatic vein interface, without compromising vascular integrity. This anatomy-preserving approach may facilitate radical resection in selected patients with vascular involvement of the future liver remnant who would otherwise be considered unresectable.

Although limited by the small sample size and short follow-up, these findings support further investigation of IRE as an adjunct to liver resection within a multimodal treatment strategy. Larger prospective studies are required to define its oncologic benefit and to clarify its role in relation to vascular reconstruction and R1-vascular resection.

## Data Availability

The raw data supporting the conclusions of this article will be made available by the authors, without undue reservation.

## References

[1] Banales JM Cardinale V Carpino G Marzioni M Andersen JB Invernizzi P. Cholangiocarcinoma: current knowledge and future perspectives consensus statement from the European network for the study of cholangiocarcinoma (ENS-CCA). Nat Rev Gastroenterol Hepatol. (2016) 13(5):261–80. 10.1038/nrgastro.2016.51 27095655

[2] Gilliaux Q Faugeras L Martinet JP De Ronde T Badaoui A Bertrand C. Incidence and prognosis of biliary tract and gallbladder cancers in a Belgian academic hospital. J Gastrointest Canc. (2021) 52(3):1003–9. 10.1007/s12029-020-00526-5 32984916

[3] Saha SK Zhu AX Fuchs CS Brooks GA. Forty-Year trends in cholangiocarcinoma incidence in the U.S.: intrahepatic disease on the rise. Oncologist. (2016) 21(5):594–9. 10.1634/theoncologist.2015-0446 27000463 PMC4861366

[4] Alvaro D Gores GJ Walicki J Hassan C Sapisochin G Komuta M. EASL-ILCA Clinical practice guidelines on the management of intrahepatic cholangiocarcinoma. J Hepatol. (2023) 79(1):181–208. 10.1016/j.jhep.2023.03.010 37084797

[5] DeOliveira ML Cunningham SC Cameron JL Kamangar F Winter JM Lillemoe KD. Cholangiocarcinoma: thirty-one-year experience with 564 patients at a single institution. Ann Surg. (2007) 245(5):755–62. 10.1097/01.sla.0000251366.62632.d3 17457168 PMC1877058

[6] Valle JW Furuse J Jitlal M Beare S Mizuno N Wasan H. Cisplatin and gemcitabine for advanced biliary tract cancer: a meta-analysis of two randomised trials. Ann Oncol. (2014) 25(2):391–8. 10.1093/annonc/mdt540 24351397

[7] Bridgewater J Galle PR Khan SA Llovet JM Park JW Patel T. Guidelines for the diagnosis and management of intrahepatic cholangiocarcinoma. J Hepatol. (2014) 60(6):1268–89. 10.1016/j.jhep.2014.01.021 24681130

[8] Moris D Palta M Kim C Allen PJ Morse MA Lidsky ME. Advances in the treatment of intrahepatic cholangiocarcinoma: an overview of the current and future therapeutic landscape for clinicians. CA Cancer J Clin. (2023) 73(2):198–222. 10.3322/caac.21759 36260350

[9] Yoh T Hatano E Yamanaka K Nishio T Seo S Taura K. Is surgical resection justified for advanced intrahepatic cholangiocarcinoma? Liver Cancer. (2016) 5(4):280–9. 10.1159/000449339 27781200 PMC5075804

[10] Olthof PB Franssen S Van Keulen AM Van Der Geest LG Hoogwater FJH Coenraad M. Nationwide treatment and outcomes of intrahepatic cholangiocarcinoma. HPB (Oxford). (2023) 25(11):1329–36. 10.1016/j.hpb.2023.06.019 37532665

[11] Conci S Viganò L Ercolani G Gonzalez E Ruzzenente A Isa G. Outcomes of vascular resection associated with curative intent hepatectomy for intrahepatic cholangiocarcinoma. Eur J Surg Oncol. (2020) 46(9):1727–33. 10.1016/j.ejso.2020.04.007 32360063

[12] Chan KM Tsai CY Yeh CN Yeh TS Lee WC Jan YY. Characterization of intrahepatic cholangiocarcinoma after curative resection: outcome, prognostic factor, and recurrence. BMC Gastroenterol. (2018) 18(1):180. 10.1186/s12876-018-0912-x 30514231 PMC6278092

[13] Farges O Fuks D Boleslawski E Le Treut YP Castaing D Laurent A. Influence of surgical margins on outcome in patients with intrahepatic cholangiocarcinoma: a multicenter study by the AFC-IHCC-2009 study group. Ann Surg. (2011) 254(5):824–30. 10.1097/SLA.0b013e318236c21d 22042474

[14] Mabilia A Mazzotta AD Robin F Ghallab M Vibert E Adam R. R1 vascular or parenchymal margins: what is the impact after resection of intrahepatic cholangiocarcinoma? Cancers (Basel). (2022) 14(20):5151. 10.3390/cancers14205151 36291934 PMC9601275

[15] Rubinsky B Onik G Mikus P. Irreversible electroporation: a new ablation modality — clinical implications. Technol Cancer Res Treat. (2007) 6(1):37–48. 10.1177/153303460700600106 17241099

[16] Brock RM Beitel-White N Davalos RV Allen IC. Starting a fire without flame: the induction of cell death and inflammation in electroporation-based tumor ablation strategies. Front Oncol. (2020) 10:1235. 10.3389/fonc.2020.01235 32850371 PMC7399335

[17] Imran KM Brock RM Beitel-White N Powar M Orr K Aycock KN. Irreversible electroporation promotes a pro-inflammatory tumor microenvironment and anti-tumor immunity in a mouse pancreatic cancer model. Front Immunol. (2024) 15:1352821. 10.3389/fimmu.2024.1352821 38711517 PMC11070574

[18] Vogel JA Van Veldhuisen E Agnass P Crezee J Dijk F Verheij J. Time-Dependent impact of irreversible electroporation on pancreas, liver, blood vessels and nerves: a systematic review of experimental studies. Rakonczay Z, éditeur. PLoS One. (2016) 11(11):e0166987. 10.1371/journal.pone.0166987 27870918 PMC5117758

[19] Narayanan G Gentile NT Eyshi J Schiro BJ Gandhi RT Peña CS. Irreversible electroporation in treating colorectal liver metastases in proximity to critical structures. J Vasc Interv Radiol. (déc 2024) 35(12):1806–13. 10.1016/j.jvir.2024.08.021 39218213

[20] Ruarus AH Vroomen LGPH Puijk RS Scheffer HJ Zonderhuis BM Kazemier G. Irreversible electroporation in hepatopancreaticobiliary tumours. Can Assoc Radiol J. (2018) 69(1):38–50. 10.1016/j.carj.2017.10.005 29458954

[21] Ma YY Shi JJ Chen JB Xu KC Niu LZ. Irreversible electroporation for liver metastasis from pancreatic cancer: a case report. World J Clin Cases. (2020) 8(2):390–7. 10.12998/wjcc.v8.i2.390 32047790 PMC7000941

[22] Yu M Li S. Irreversible electroporation for liver cancer ablation: a meta analysis. Eur J Surg Oncol. (2022) 48(6):1321–30. 10.1016/j.ejso.2021.12.015 35012834

[23] Belfiore MP Reginelli A Maggialetti N Carbone M Giovine S Laporta A. Preliminary results in unresectable cholangiocarcinoma treated by CT percutaneous irreversible electroporation: feasibility, safety and efficacy. Med Oncol. (2020) 37(5):45. 10.1007/s12032-020-01360-2 32270353

[24] Sweeney J Parikh N El-Haddad G Kis B. Ablation of intrahepatic cholangiocarcinoma. Semin Intervent Radiol. (2019) 36(04):298–302. 10.1055/s-0039-1696649 31680720 PMC6823041

[25] Martin RCG Kwon D Chalikonda S Sellers M Kotz E Scoggins C. Treatment of 200 locally advanced (stage III) pancreatic adenocarcinoma patients with irreversible electroporation: safety and efficacy. Annals of Surgery. (2015) 262(3):486–94. 10.1097/SLA.0000000000001441 26258317

[26] Patel A Lancellotti F Siriwardena AK Nadarajah V De Liguori Carino N, HPB-MRI Collaborative Group. Irreversible electroporation as an intraoperative adjunctive treatment for locally advanced pancreatic cancer after neoadjuvant therapy: an initial clinical experience. Ann Hepatobiliary Pancreat Surg. (2025) 29(1):72–8. 10.14701/ahbps.24-193 39819732 PMC11830891

[27] Ratnayake B Al-Leswas D Mohammadi-Zaniani G Littler P Sen G Manas D. Margin accentuation irreversible electroporation in stage III pancreatic cancer: a systematic review. Cancers (Basel). (2021) 13(13):3212. 10.3390/cancers13133212 34199031 PMC8268790

[28] Kwon D McFarland K Velanovich V Martin RCG. Borderline and locally advanced pancreatic adenocarcinoma margin accentuation with intraoperative irreversible electroporation. Surgery. (2014) 156(4):910–22. 10.1016/j.surg.2014.06.058 25239345

[29] Tian G Zhao Q Chen F Jiang T Wang W. Ablation of hepatic malignant tumors with irreversible electroporation: a systematic review and meta-analysis of outcomes. Oncotarget. (2017) 8(4):5853–60. 10.18632/oncotarget.14030 28009979 PMC5351595

[30] Owen M Makary MS Beal EW. Locoregional therapy for intrahepatic cholangiocarcinoma. Cancers (Basel). (2023) 15(8):2384. 10.3390/cancers15082384 37190311 PMC10137284

[31] Chapelle T Op De Beeck B Huyghe I Francque S Driessen A Roeyen G. Future remnant liver function estimated by combining liver volumetry on magnetic resonance imaging with total liver function on 99mTc-mebrofenin hepatobiliary scintigraphy: can this tool predict post-hepatectomy liver failure? HPB (Oxford). (2016) 18(6):494–503. 10.1016/j.hpb.2015.08.002 27317953 PMC4913132

[32] Ruarus AH Vroomen LGPH Puijk RS Scheffer HJ Faes TJC Meijerink MR. Conductivity rise during irreversible electroporation: true permeabilization or heat? Cardiovasc Intervent Radiol. (2018) 41(8):1257–66. 10.1007/s00270-018-1971-7 29687261 PMC6021471

[33] Dunki-Jacobs EM Philips P Martin RCG. Evaluation of resistance as a measure of successful tumor ablation during irreversible electroporation of the pancreas. J Am Coll Surg. (2014) 218(2):179–87. 10.1016/j.jamcollsurg.2013.10.013 24315888

[34] Dindo D Demartines N Clavien PA. Classification of surgical complications: a new proposal with evaluation in a cohort of 6336 patients and results of a survey. Ann Surg. (2004) 240(2):205–13. 10.1097/01.sla.0000133083.54934.ae 15273542 PMC1360123

[35] Rahbari NN Garden OJ Padbury R Maddern GJ Koch M Hugh TJ. Posthepatectomy liver failure: a definition and grading by the international study group of liver surgery (ISGLS). Surgery. (2011) 149:713–24. 10.1016/j.surg.2010.10.001 21236455

[36] Nagino M DeMatteo R Lang H Cherqui D Malago M Kawakatsu S. Proposal of a new comprehensive notation for hepatectomy: the “new world” terminology. Ann Surg. (2021) 274(1):1–3. 10.1097/SLA.0000000000004808 33630445

[37] Hu LS Zhang XF Weiss M Popescu I Marques HP Aldrighetti L. Recurrence patterns and timing courses following curative-intent resection for intrahepatic cholangiocarcinoma. Ann Surg Oncol. (2019) 26(8):2549–57. 10.1245/s10434-019-07353-4 31020501

[38] Langella S Russolillo N Ossola P Luzzi AP Casella M Lo Tesoriere R. Recurrence after curative resection for intrahepatic cholangiocarcinoma: how to predict the chance of repeat hepatectomy? J Clin Med. (2021) 10(13):2820. 10.3390/jcm10132820 34206799 PMC8269164

[39] Milana F Procopio F Calafiore E Famularo S Costa G Galvanin J. Long-Term outcomes according to surgical margin in mass-forming cholangiocarcinoma: the role of R1vasc. Ann Surg Oncol. (2025) 32(6):4363–73. 10.1245/s10434-025-17038-w 40019600

[40] Shimoda M Farmer DG Colquhoun SD Rosove M Ghobrial MR Yersiz H. Liver transplantation for cholangiocellular carcinoma: analysis of a single-center experience and review of the literature. Liver Transpl. (2001) 7(12):1023–33. 10.1053/jlts.2001.29419 11753904

[41] Ziogas IA Giannis D Economopoulos KP Hayat MH Montenovo MI Matsuoka LK. Liver transplantation for intrahepatic cholangiocarcinoma: a meta-analysis and meta-regression of survival rates. Transplantation. (2021) 105(10):2263–71. 10.1097/TP.0000000000003539 33196623

[42] Miyata A Sakamoto Y Yamamoto S Akamatsu N Arita J Kaneko J. Aggressive hemihepatectomy combined with resection and reconstruction of middle hepatic vein for intrahepatic cholangiocarcinoma. Ann Surg Oncol. (2016) 23(S4):494–500. 10.1245/s10434-016-5384-z 27384750

[43] Sadamori H Hasegawa K Oba A Kato Y Soejima Y Monden K. Short- and long-term outcomes of liver resection with hepatic vein reconstruction for liver tumors: a nationwide multicenter survey. J Hepato Biliary Pancreat. (2024) 31(12):863–75. 10.1002/jhbp.12077 39474884

[44] Viganò L Procopio F Cimino MM Donadon M Gatti A Costa G. Is tumor detachment from vascular structures equivalent to R0 resection in surgery for colorectal liver metastases? An observational cohort. Ann Surg Oncol. (2016) 23(4):1352–60. 10.1245/s10434-015-5009-y 26714946

[45] Donadon M Terrone A Procopio F Cimino M Palmisano A Viganò L. Is R1 vascular hepatectomy for hepatocellular carcinoma oncologically adequate? Analysis of 327 consecutive patients. Surgery. (2019) 165(5):897–904. 10.1016/j.surg.2018.12.002 30691871

[46] Torzilli G Viganò L Fontana A Procopio F Terrone A Cimino MM. Oncological outcome of R1 vascular margin for mass-forming cholangiocarcinoma. A single center observational cohort analysis. HPB (Oxford). (2020) 22(4):570–7. 10.1016/j.hpb.2019.08.015 31530450

[47] Maspero M Sposito C Bongini MA Cascella T Flores M Maccauro M. Liver transplantation for intrahepatic cholangiocarcinoma after chemotherapy and radioembolization: an intention-to-treat study. Transpl Int. (2024) 37:13641. 10.3389/ti.2024.13641 39544321 PMC11560448

[48] Oh DY Ruth He A Qin S Chen LT Okusaka T Vogel A. Durvalumab plus gemcitabine and cisplatin in advanced biliary tract cancer. NEJM Evid. (2022) 1(8):1–11. 10.1056/EVIDoa2200015 38319896

[49] Edeline J Touchefeu Y Guiu B Farge O Tougeron D Baumgaertner I. Radioembolization plus chemotherapy for first-line treatment of locally advanced intrahepatic cholangiocarcinoma: a phase 2 clinical trial. JAMA Oncol. (2020) 6(1):51. 10.1001/jamaoncol.2019.3702 31670746 PMC6824230

[50] Edeline J Rolland Y Campillo-Gimenez B Bourien H Trochet M Garin E. Selective internal radiation therapy (yttrium-90 glass microspheres) combined with capecitabine in the neoadjuvant setting of operable intrahepatic CHOlangiocarcinoma: the SIROCHO trial design. Ann Surg Oncol. (2023) 30(1):21–2. 10.1245/s10434-022-12708-5 36271306

[51] Adamus N Edeline J Henriques J Fares N Lecomte T Turpin A. First-line chemotherapy with selective internal radiation therapy for intrahepatic cholangiocarcinoma: the French ACABi GERCOR PRONOBIL cohort. JHEP Reports. (2025) 7(2):101279. 10.1016/j.jhepr.2024.101279 39897613 PMC11786833

[52] Teo JY Allen JC Ng DC Choo SP Tai DWM Chang JPE. A systematic review of contralateral liver lobe hypertrophy after unilobar selective internal radiation therapy with Y90. HPB (Oxford). (2016) 18(1):7–12. 10.1016/j.hpb.2015.07.002 26776845 PMC4750235

[53] Palloni A Bisello S Maggio I Massucci M Galuppi A Di Federico A. The potential role of adjuvant chemoradiotherapy in patients with microscopically positive (R1) surgical margins after resection of cholangiocarcinoma. Current Oncology. (2023) 30(5):4754–66. 10.3390/curroncol30050358 37232816 PMC10217181

[54] Zhang Y Huang ZX Song B. Role of imaging in evaluating the response after neoadjuvant treatment for pancreatic ductal adenocarcinoma. World J Gastroenterol. (2021) 27(22):3037–49. 10.3748/wjg.v27.i22.3037 34168406 PMC8192284

[55] Martin RCG Schoen EC Philips P Egger ME McMasters KM Scoggins CR. Impact of margin accentuation with intraoperative irreversible electroporation on local recurrence in resected pancreatic cancer. Surgery. (2023) 173(3):581–9. 10.1016/j.surg.2022.07.033 36216618 PMC9918678

[56] Ben-David E Appelbaum L Sosna J Nissenbaum I Goldberg SN. Characterization of irreversible electroporation ablation in *in vivo* porcine liver. Am J Roentgenol. (2012) 198(1):W62–8. 10.2214/AJR.11.6940 22194517

[57] Appelbaum L Ben-David E Faroja M Nissenbaum Y Sosna J Goldberg SN. Irreversible electroporation ablation: creation of large-volume ablation zones in *in vivo* porcine liver with four-electrode arrays. Radiology. (2014) 270(2):416–24. 10.1148/radiol.13130349 24126371

